# Application of unsupervised learning to cluster swine breeding herds based on key performance indicators in Southern Brazil

**DOI:** 10.1093/tas/txag036

**Published:** 2026-03-25

**Authors:** Rafael R Ulguim, Marcelo Alexandrino Pereira, Julia Tavares, Daniel C L Linhares, Ana Paula G Mellagi, Fernando P Bortolozzo, Cícero Ari Tecchio, Ricardo Yuiti Nagae, Neimar Cristiano Cavazini, Anne Caroline de Lara, Gustavo S Silva

**Affiliations:** Setor de Suínos—Departamento de Medicinal Animal, Universidade Federal do Rio Grande do Sul, Av. Bento Gonçalves, Porto Alegre, RS, 91540-000, Brazil; Setor de Suínos—Departamento de Medicinal Animal, Universidade Federal do Rio Grande do Sul, Av. Bento Gonçalves, Porto Alegre, RS, 91540-000, Brazil; Setor de Suínos—Departamento de Medicinal Animal, Universidade Federal do Rio Grande do Sul, Av. Bento Gonçalves, Porto Alegre, RS, 91540-000, Brazil; FieldEpi—Department of Veterinary Diagnostic and Production Animal Medicine, Iowa State University, Lloyd Veterinary Medical Center, Ames, IA, 50011, United States; Setor de Suínos—Departamento de Medicinal Animal, Universidade Federal do Rio Grande do Sul, Av. Bento Gonçalves, Porto Alegre, RS, 91540-000, Brazil; Setor de Suínos—Departamento de Medicinal Animal, Universidade Federal do Rio Grande do Sul, Av. Bento Gonçalves, Porto Alegre, RS, 91540-000, Brazil; Seara Alimentos Ltda, Rodovia Antônio Heil, Itajaí, SC, 88316-003, Brazil; Seara Alimentos Ltda, Rodovia Antônio Heil, Itajaí, SC, 88316-003, Brazil; Seara Alimentos Ltda, Rodovia Antônio Heil, Itajaí, SC, 88316-003, Brazil; Seara Alimentos Ltda, Rodovia Antônio Heil, Itajaí, SC, 88316-003, Brazil; FieldEpi—Department of Veterinary Diagnostic and Production Animal Medicine, Iowa State University, Lloyd Veterinary Medical Center, Ames, IA, 50011, United States

**Keywords:** sow farm, reproductive performance, classification of farms, cluster analysis

## Abstract

Reproductive performance in sow farms has improved over time in most production systems. However, variability among farms remains substantial. This study employed unsupervised analysis to cluster farms based on reproductive indicators and assess the farm-level characteristics associated with performance differences. A total of 22 breeding herds in the Southern Brazil region were surveyed to gather demographic, labor, infrastructure, environmental, management, and production data. Monthly reproductive performance indicators from 2022 and 2023 were collected, including farrowing rate (FR), total piglets born (TPB), piglets born alive (TBA), stillborn (SB), and pre-weaning mortality (PWM). A K-means clustering analysis grouped farms based on five key reproductive performance indicators (KPIs). Cluster identification was used as an independent variable to identify differences in survey responses. The unsupervised model suggested two clusters based on the consensus of 26 indexes. The high-performance cluster (Cluster 2, *n* = 12) exhibited a higher FR (+3.6%), lower SB (−1.6%), and PWM (−1.7%) compared to the low-performance cluster (Cluster 1, *n* = 10) (*P* ≤ 0.01). No significant differences were found between clusters in labor characteristics (*P* ≥ 0.24). However, the high-performance cluster had a lower percentage of farms using farrowing induction in gilts, a reduced percentage of re-serviced sows included in the breeding groups, fewer farrowing crates per room, and a higher rate of farms introducing creep-feeding earlier during lactation (*P* ≤ 0.05). This study demonstrates the effectiveness of an unsupervised clustering approach in classifying breeding herds based on five KPIs. This method can identify key farm-level factors that distinguish herd performance, offering insights for improving swine reproductive performance.

## Teaser

This study provides data-driven insights that can help farms optimize productivity and piglet survival by identifying key management practices linked to high reproductive performance swine herds. The findings demonstrate how clustering analysis can be a tool to identify opportunities for improving swine breeding herd efficiency.

## Introduction

The reproductive performance of swine breeding herds exhibits considerable variability, influenced by multiple factors, including genetics, nutrition, environmental conditions, and management strategies (Knox 2016; Koketsu et al 2017). Such variability is particularly evident when analyzing key production indicators (KPI), such as piglets weaned per sow per year (PSY), which varies significantly between the industry average and the average of the top 5 farms (30.0 and 38.1 PSY, respectively). A similar pattern is observed for farrowing rate, which ranges from 87.3% to 93.8% in the Brazilian pig industry (Agriness 2025). Understanding this variation requires a holistic evaluation, integrating both intrinsic biological aspects at the individual animal level and extrinsic influences such as farm infrastructure and management practices (Koketsu 2005; Koketsu et al 2017; Koketsu and Iida 2020; Sanz-Fernández et al 2024; Knox 2024). Various approaches have been explored to enhance reproductive efficiency, including modifications in housing systems, management changes, and benchmarking initiatives. While these strategies contribute to improvements, they also reinforce the need for advanced analytical techniques to distinguish productivity differences between high- and low-performing herds.

Regression models have been extensively used to evaluate the association between sow- and herd-level factors and reproductive outcomes. Iida et al (2015) and Tani et al (2016) applied multi-level regression models to evaluate variables such as farrowing rate and pigs born alive (PBA). Their findings highlighted key predictors of reproductive performance, including extreme parities, inadequate lactation feed intake, prolonged weaning-to-first-mating intervals, and high ambient temperatures. Similarly, regression techniques to assess management practices for prolific sows under tropical conditions (Tummaruk et al 2010) emphasize the detrimental effects of season and variability among herds on farrowing rate. Additionally, seasonal variations, specific productivity variables, health status, and farm infrastructure were identified as risk factors associated with piglet pre-weaning mortality (Will et al 2024). However, while regression models reveal significant associations between specific risk factors and reproductive indicators, they do not inherently classify herds based on productivity levels.

Classification methods based on quartiles or percentiles of KPI have been traditionally applied to better characterize performance among breeding herds, particularly using individual KPI such as PSY (Koketsu 2000, 2007). High-performing herds exhibit superior operational strategies, including more efficient mating schedules, reduced non-productive days, and improved farrowing rates. Comparative studies have also identified management practices linked to enhanced productivity, such as strategic culling of low-efficiency sows at early parities, which helps minimize reproductive inefficiencies (Koketsu 2007). Although helpful in classifying herds, percentile-based selection is typically centered on a single productivity indicator, which may overlook interdependencies among performance drivers.

Cluster analysis emerges as an alternative classification method, enabling the segregation of herds from patterns in productivity indicators. Unlike percentile-based classification, clustering identifies natural groupings of herds by analyzing patterns across multiple KPI. While cluster analysis does not inherently assess multivariate interactions or risk factor relationships, its application facilitates the segmentation of herds into distinct clusters based on patterns in productivity indicators. Combining mechanistic and data-driven models improves precision agriculture, particularly for optimizing predictive accuracy and system-level understanding (Ellis et al 2020). Machine learning techniques also contribute significantly to decision-making processes in animal production systems (van Klompenburg and Kassahun 2022). Considering these perspectives, cluster analysis represents an additional tool for refining herd classification methods and optimizing management strategies. To the best of our knowledge, no previous studies in the literature have applied this approach to segregate herds using different sow farm KPIs together.

This study hypothesizes that clustering techniques applied to swine herds based on multiple KPI can facilitate herd classification and provide insights into farm-level factors associated with performance differences. The objective was to classify breeding herds using selected reproductive KPI within an unsupervised framework to identify farm-level characteristics that drive variations in reproductive performance among breeding herds.

## Materials and methods

This study was approved under protocol number 46298 by the Federal University of Rio Grande do Sul Scientific Research Committee.

### Study design and farm characteristics survey

An observational study was conducted across 22 breeding herds in the Rio Grande do Sul State, Southern Brazil. The present study used only breeding herds from the same company with the same genetics (Agroceres PIC Camborough^®^ 1050). A total of 22 farms (average—1277 sows/farm; min—330; max—3024) with a total inventory of 28,096 sows were used. All farms were located between latitudes 28°S and 29°S. The eligibility criteria for including farms in this study were: a breeding herd with no changes or fluctuations in herd size, production flow, and infrastructure between 2020 and 2024. All herds belonged to the same health pyramid, being positive for *Mycoplasma hyopneumoniae* and free of PRRS.

All farms were visited, and eight technicians were trained to conduct an on-farm survey. The technicians were trained to evaluate the infrastructure and perform an interview with the farm owner or farm manager using a survey. The survey included questions (Table S1) to gather information about each farm’s demographics, labor, infrastructure, environment, management practices, and productive data. The survey was conducted on farms between January and August 2024. In all farms, farrowings were supervised throughout all working shifts and farrowing induction performed with a minimum of 1 d before the expected farrowing date. Among farms adopting pen gestation, the majority (80%) mixed sows approximately 30 d after insemination. The production data were collected through a commercial record-keeping system software used by the company (Agriness S4, Brazil). Monthly reproductive performance indicators like farrowing rate (FR), the total number of piglets born (TPB), piglets born alive (TBA), stillborn (SB), and pre-weaning mortality (PWM) from 2022 and 2023 were pulled from the software reports, along with monthly percentages of reserviced sows, gilts and sows breed.

**Table 1:** Farm traits and reproductive performance between clusters after unsupervised classification of 22 breeding herds based on five key reproductive indicators.

Variable	**Cluster** ^a^		*P*-value
	Low-performance	High-performance	
***n***	10	12	
**Farm age, years**	26.5 (23.0; 29.5)	24.5 (23.0; 30.8)	0.82
**Average herd size**	1221.5 (735.0; 1484.8)	1321.0 (605.0; 1500.5)	0.92
**Farrowing rate, %**	86.7 (85.6; 88.5)	90.7 (89.3; 92.3)	<0.01
**Total piglets born**	15.7 (15.4; 15.9)	15.4 (15.3; 15.7)	0.21
**Total piglets born alive**	14.5 (14.1; 14.7)	14.5 (14.3; 14.7)	0.55
**Stillborn piglets, %**	5.8 (5.6; 6.1)	4.2 (3.7; 4.5)	<0.01
**Preweaning mortality, %**	6.9 (6.3; 7.5)	5.5 (4.6; 6.3)	0.01
**Piglets weaned/1000 served sows**	11,595.5 (11,465.2; 11,729.8)	12,416.5 (12,053.8; 12,923.4)	<0.01

a Results are presented as the median and 1^st^ and 3^rd^ quantile between parentheses.

### Statistical analyses

#### Cluster analysis

A K-means cluster analysis was performed to group the farms based on five reproductive KPI: FR, TPB, TBA, SB, and PWM. The yearly average reproductive KPI during 2022 and 2023 for each indicator by farm was used. The KPIs were standardized using the Z-score calculation, and these values were used to compute the Euclidean matrix distance. The K-means clustering analysis was performed using the Euclidean matrix distance and the Ward-D2 method. The function NbClust in the R package *NbClust* was used objectively to determine the number of clusters. This function minimizes the within-cluster variance and maximizes the between-cluster variance, using 26 indices to determine the optimal number of clusters.

#### Comparison between clusters

Thereafter, statistical analyses were performed to assess differences in the survey responses and cluster groups. Continuous variables that followed a normal distribution were analyzed using a linear regression model, while non-normal distributions were analyzed using the Kruskal-Wallis test to assess differences between clusters. Due to the lack of normality in most continuous variables, values are presented as median, first quartile (q1), and third quartile (q3). The categorical variables that characterize the farms were considered binary responses (yes/no) and analyzed using Fisher’s exact test. The clusters were denominated as low- and high-performance based on the number of piglets weaned per 1000 inseminated females. All analyses were performed using Stats, *NbClust* R and *DescTools* packages in R software (R, v.4.2.3).

## Results

### Descriptive analysis of the farm’s reproductive performance

**Figure 1:**
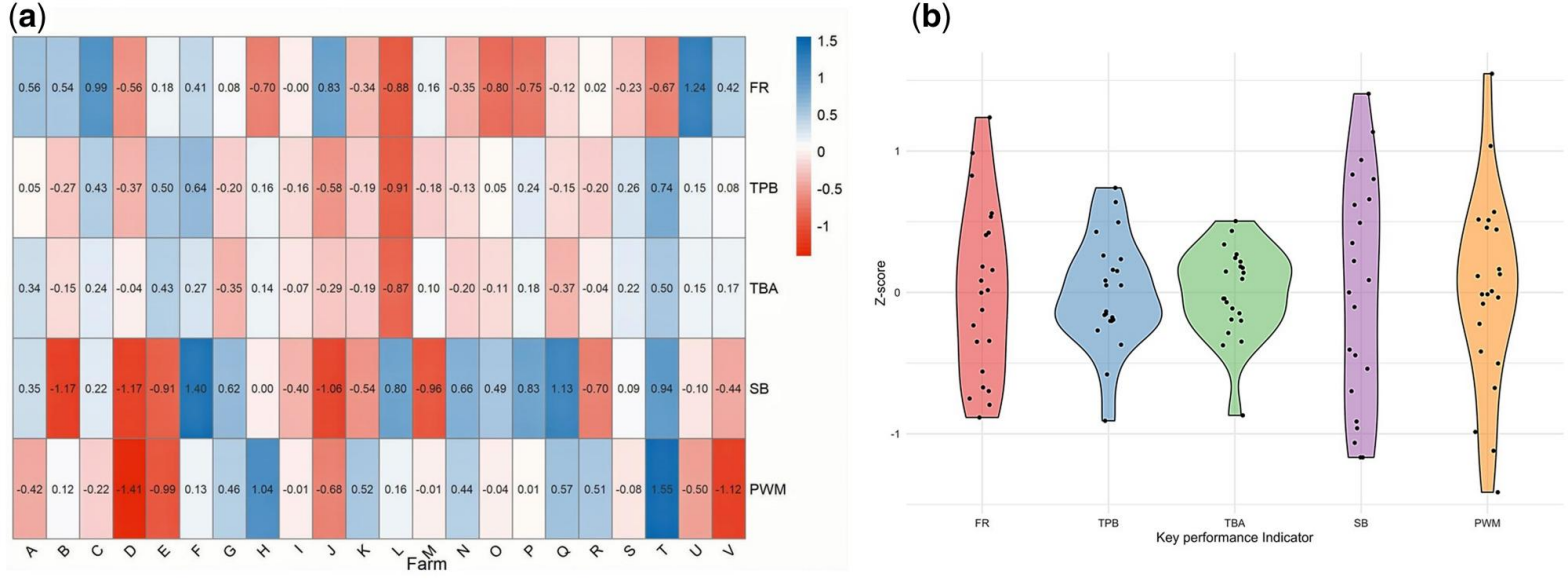
Variability on key performance indicators in breeding herds used to define the clusters for each farm a) and among farms b). Data for all indicators were standardized and presented as Z-scores. A value of zero represents the overall mean, and positive or negative values indicate the number of standard deviations above or below the mean. FR, farrowing rate; TBP, total number of piglets born; TBA, total number of piglets born alive; SB, stillborn; PWM, pre-weaning mortality

The overall reproductive performance to define the cluster for the 22 farms included in the study was: farrowing rate (mean: 89.2%; min: 84.8%; max: 95.3%); total piglets born (mean: 15.6; min: 14.5; max: 16.4); total piglets born alive (mean: 14.5; min: 13.5; max: 15.0); stillborn piglets (mean: 4.9%; min: 3.5%; max: 6.7%) and; preweaning mortality (mean: 6.25%; min: 3.1%; max: 9.7%). In Fig. 1a, variability in the reproductive performance of each farm is presented using standardized Z-scores, allowing comparison of each farm relative to the overall mean for each indicator. The red and blue colors define the farms with results below or above the average (defined as 0) for each indicator, respectively. This descriptive result indicates that, in general, 40.9% (9/22) of the farms had a variation of higher than 0.5 standard deviations above or below the average for FR, SB, and PWM. Higher variability was observed for SB, with 59.1% (13/22) of farms exceeding ±0.5 standard deviations from the mean. In contrast, lower variability was observed for TBA, with 4.5% (1/22) of farms exceeding ±0.5 standard deviation relative to the mean. Additionally, 31.8% (7/22) of the farms performed better than the overall mean in four or more reproductive indicators, while 13.6% (3/22) showed superior results in no more than one indicator. The remaining 54.5% (12/22) had just two or three indicators exceeding the average performance level. The variability for each indicator can be observed through the distribution spread in the violin plots (Fig. 1b). A wider spread indicates more significant variability for FR, SB, and PWM, while a narrower shape suggests lower dispersion for TPB and TBA.

### Cluster analysis

**Figure 2:**
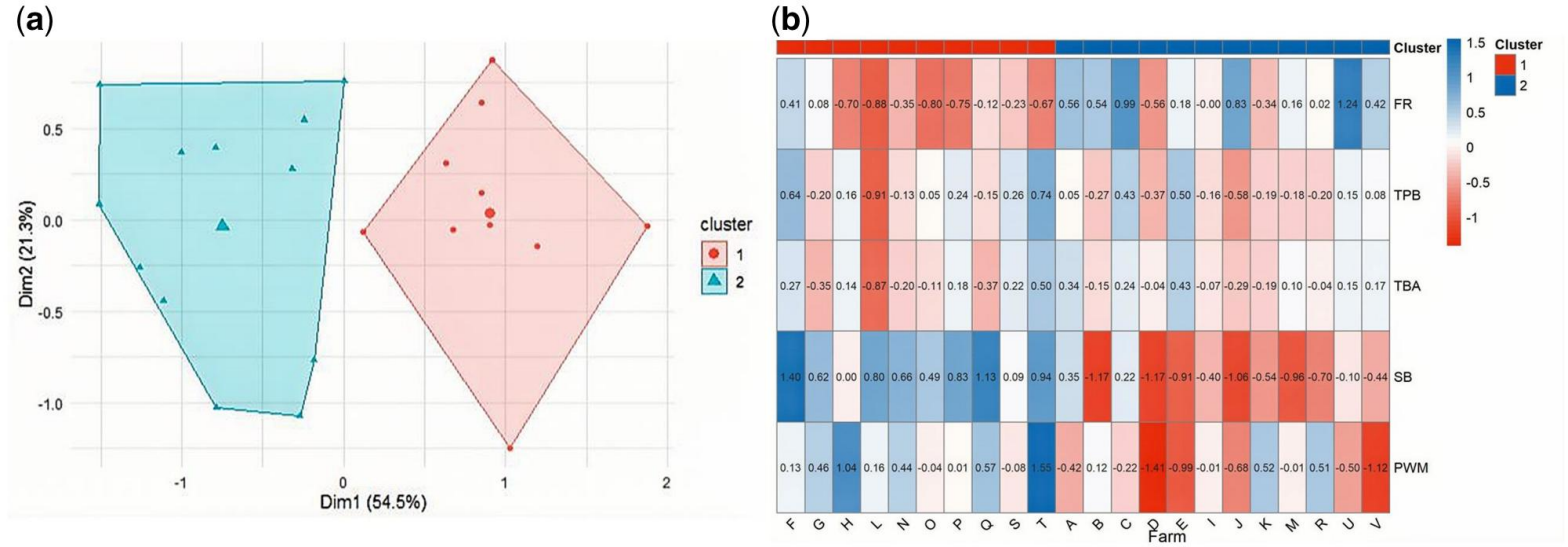
Cluster analysis a) is based on five key performance indicators in sow farms from Rio Grande Do Sul (Brazil) and the distribution of farms between clusters b). Cluster 1 represents the “low reproductive performance farms,” and cluster 2 is “high reproductive performance farms.”

The unsupervised model suggested two clusters that were well segregated from each other (Fig. 2a). Based on the differences in the centroids, the most important variables in defining the clusters were farrowing rate, stillborn, and preweaning mortality. The distribution of farms in each cluster is presented in Fig. 2b. There were no differences in the farm’s age or number of sows in the inventory between clusters (*P* ≥ 0.82). Cluster 2 presented higher FR, lower SB, and PWM compared to Cluster 1. Based on this response, the clusters were denominated as high (cluster 2) and low (cluster 1) reproductive performance (Table 1). The high-performance cluster resulted, on average, in 827.3 more weaned piglets per 1000 serviced sows than the low-performance cluster (Table 1; *P* ≤ 0.01).

There was no difference between clusters on reported labor characteristics gathered from farms included in this study. Additionally, it is important to highlight that housing sows in gestation pens did not differ between clusters. Among 26 infrastructure and handling characteristics in the gestating sector, four variables were significant or tended to differentiate high- and low-performance herds. In the farrowing unit, three characteristics related to infrastructure and handling, from 10 variables collected, were significant or tended to differentiate high- and low-performance herds. The percentage of farms using farrowing induction in gilts was markedly lower (*P* = 0.03) in the high-performance cluster (25%) compared with the low-performance cluster (80%; Table 2). Furthermore, the average number of farrowing crates per room was substantially lower (*P* = 0.02; Table 3) in the high-performance cluster (53 crates/room) compared to the low-performance cluster (156 crates/room).

**Table 2:** Categorical variables gathered from farms with different characteristics in infrastructure, labor, and handling according to low- and high-reproductive performance clusters.

Farm characteristic	Cluster, %^a^		*P*-value^b^
	Low-performance	High-performance	
***n***	10	12	
***General characteristics of the farms***			
** Batch flow**			0.18
** 1** **wk**	0.0	8.3	
** 2** **wk**	20.0	50.0	
** 3** **wk**	20.0	0.0	
** 4** **wk**	60.0	41.7	
** Another activity on the farm (dairy farm, agriculture, etc.)**	80.0	50.0	0.20
** Presence of a power generator**	60.0	50.0	0.69
** Barn orientation (East-West)**	50.0	66.7	0.67
** Water tank maintained in the shadow**	30.0	63.6	0.20
***Labor***			
** Farm labor provided by owners**	20.0	33.3	0.65
** Farms with >50% of workers with more than 5** **yr in the farm**	80.0	91.7	0.57
** Farms with >50% of workers with more than 5** **yr in the gestation sector**	30.0	58.3	0.24
** Farms with >50% of workers with more than 5** **yr in the farrowing sector**	70.0	75.0	1.00
** Monthly workers’ training on protocols**	40.0	41.7	1.00
***Gestating infrastructure and handling***			
** Cooling system in the gestation barn**	20.0	0	0.19
** Presence of grass and trees around the gestation barn**	50.0	75.0	0.38
** Use of bump-feeding strategy during gestation**	50.0	16.7	0.17
** Automated barn lighting control**	50.0	16.7	0.17
** Individual water nipples while in gestation stalls**	10.0	50.0	**0.07**
** Floor feeding while in gestation stalls**	100	75.0	0.22
** Gestating sows housed in pens**	60.00	41.7	0.67
** Use of antimicrobials in gilts at the entrance**	40.0	50.0	0.97
** Acclimation of gilts at the entrance using some exposure method**	10.0	33.3	0.32
** Use of enteric and/or reproductive feedback in gilts**	20.0	50.0	0.20
** Use of enteric and/or reproductive feedback in sows**	10.0	33.3	0.32
** Sows housed in pens based on parity**	50.0	60.0	1.00
** Sows housed in pens based on BCS**	100	80.0	0.45
***Farrowing infrastructure and handling***			
** Different types of farrowing barn infrastructure**	40.0	58.3	0.67
** Presence of grass and trees around the farrowing barn**	40.0	75.0	0.19
** Cooling system in the farrowing barn**	20.0	16.7	1.00
** Heat system in farrowing barn**	90.0	75.0	0.59
** Farrowing induction procedure**	100	100	–
** Farrowing induction in gilts**	80.0	25.0	**0.03**
** Encourage sows to stand during lactation at least once a day**	70.0	58.3	0.67

a Represents the percentage of farms within each category of variable.

b Bold values indicate statistically significant (P < 0.05) or tendency (0.05 ≤ P < 0.10).

**Table 3:** Continuous variables^a^ for different characteristics in infrastructure, labor, and handling according to low- and high-performance cluster classification.

Farm characteristic	Cluster		*P*-value^b^
	Low-performance	High-performance	
***n***	10	12	
***General characteristics of the farms***			
** Farm distance from the boar stud, km**	80.0 (74.3; 95.0)	83.5 (67.5; 102.5)	0.87
** Dirt road length for semen delivery, km**	15.0 (15.0; 20.0)	17.5 (10.0; 20.0)	0.74
***Labor***			
** Number of sows/worker**	147.0 (112.8; 155.7)	127.6 (105.7; 133.8)	0.47
** Percentage of workers with ≥5** **yr on the farm**	42.2 (20.3; 87.5)	59.1 (20.8; 87.5)	0.62
** Percentage of workers with ≥5** **yr in the gestation sector**	22.2 (12.5; 24.5)	20.0 (17.2; 22.9)	0.82
** Percentage of workers with ≥5** **yr in the farrowing sector**	77.8 (75.0; 87.5)	79.1 (74.1; 80.5)	0.87
***Gestating infrastructure and handling***			
** Number of gilts per pen during puberty induction**	10.0 (7.0; 15.0)	10.5 (9.8; 12.0)	0.91
** Days in stalls before breeding the gilts, days**	15.5 (15.0; 20.0)	15.0 (15.0; 15.0)	0.33
** Number of sows in the breeding group**	195.0 (132.5; 207.5)	117.5 (100.0; 130.0)	**0.08**
** Percentage of re-serviced sows in the breeding groups**	6.5 (5.9; 6.9)	4.2 (3.4; 5.2)	**<0.01**
** Percentage of gilts in the breeding groups**	21.5 (19.8; 22.6)	20.5 (19.4; 21.6)	0.53
** Number of sows per teaser boars**	208.4 (180.4; 277.8)	204.6 (166.9; 278.8)	0.77
** Time spent for estrus detection, min/50 female**	19.6 (15.2; 22.9)	24.5 (20.2; 45.3)	0.11
** Number of gestating sows per collective pen**	20.0 (10.0; 22.0)	20.0 (15.0; 24.5)	0.76
** Percentage of the compact floor in pen gestation**	40.0 (32.5; 40.0)	30.0 (25.0; 40.0)	0.44
** Number of sows per water nipple in collective pens**	10.5 (10.0; 11.0)	8.0 (7.0; 10.0)	**0.07**
***Farrowing infrastructure and handling***			
** Number of farrowing crates per room**	156.0 (85.0; 194.5)	53.0 (28.0; 101.0)	**0.02**
** Day post-farrowing to start creep-feeding for piglets**	5.0 (5.0; 7.0)	5.0 (4.5; 5.0)	**0.05**

a Results are presented as the median and 1^st^ and 3^rd^ quantiles between parentheses.

b Bold values indicate statistically significant (P < 0.05) or tendency (0.05 ≤ P < 0.10).

Additionally, a higher percentage of farms started creep-feeding for piglets before the fifth day of lactation in the high-performance cluster (Table 3). The rate of re-serviced sows included in the breeding groups was 2.3% lower for the high-performance (Table 3) compared to low-performance herds (*P* < 0.01). A tendency (*P* ≤ 0.08) was observed for a higher percentage of farms in the high-performance herds to adopt individual water nipples for sows housed in gestation stalls (Table 2), as well as a lower number of sows per water nipple in collective gestation pens (Table 3), when compared to low-performance herds.

## Discussion

Most published studies have traditionally grouped swine breeding herds based on productivity using a single KPI, often using percentiles or quartiles for classification. In contrast, our study incorporated multiple KPIs within an unsupervised selection framework to segregate herds based on overall productivity. The results demonstrated the effectiveness of this clustering approach in differentiated breeding herds, with the high-performance cluster exhibiting superior farrowing rates (FR), stillborn rates (SB), and pre-weaning mortality (PWM), ultimately leading to a higher number of piglets weaned per 1000 served sows. Furthermore, this methodology enabled the identification of key farm-level drivers associated with performance differences.

One notable finding was that the high-performance cluster had fewer farrowing crates per room than the low-performance cluster. To our knowledge, the relationship between the number of farrowing crates per room and its impact on SB or PWM has not been extensively explored in the literature. However, from a practical standpoint, fewer crates per room may facilitate individualized sow and piglet management, reducing SB and PWM and improving FR in high-performance clusters. Interestingly, the number of sows/worker did not differ between clusters, but the number of sows within breeding groups tended to be lower in the high-performance herds. While breeding group size is often correlated with herd size, the overall herd size remained similar across clusters. Large herd sizes (2001 to 8000 sows) have been reported to achieve higher farrowing rates compared to small (<500 sows) or medium-sized (501 to 2000 sows) farms, despite breeding larger batches and operating with a lower personnel-to-sow ratio (Knox et al 2013). However, previous studies also associate larger herd sizes with increased PWM (Koketsu et al 2006; Nuntapaitoon and Tummaruk 2018).

Conversely, Koketsu et al (2021) observed higher PWM in small to mid-sized herds compared to large herds, highlighting the variability of findings on herd size effects. Most studies focus on total herd size rather than the specific size of breeding groups, despite the shift in recent years toward batch farrowing flows with different intervals. Further studies should consider breeding or farrowing group size as a critical analytical parameter rather than solely examining herd size. In our study, the type of batch farrowing did not differ between clusters, although high-performance clusters had a numerically lower percentage of farms using 3- to 4-wk batch farrowing intervals. This distinction partially explains the differences in breeding group sizes despite similar overall herd sizes between clusters.

Efforts to meet the target of sows in the breeding batches often involve re-service sows that experienced reproductive failure (eg abortion or return to estrus). However, females with prior reproductive failure are 3.2 times more likely to return to estrus than first-service sows (Vargas et al 2009a), and their farrowing rate is significantly lower by 9.7% and 4.8% for abortion and estrus-returned sows, respectively, compared to first-served sows (Vargas et al 2009b). Koketsu (2000) similarly indicated that high-performing herds had lower percentages of re-serviced sows. Our findings reinforce this response, with high-performance herds achieving better FR and including fewer re-serviced sows within breeding groups. Farms with lower FR typically have more reproductive failures, leading to an increased reliance on rebreeding these sows. This situation perpetuates a cycle that gradually impairs herd productivity. A 5-yr study showed that high-performing herds progressively reduced the percentage of re-serviced females, whereas low-performing herds maintained stable rebreeding rates over time (Koketsu 2007). Moreover, low-performing herds exhibited a repeat breeder risk that was 0.73% to 1.19% higher than that of high-performance herds (Tani et al 2017). Although no established guidelines specify a maximum threshold for the inclusion of re-serviced sows within the breeding groups, our findings suggest keeping this percentage below 5%. Alternatively, gilts or weaned sows with good clinical conditions can replace these sows to meet breeding targets. Strict culling policies, such as those implemented in high-performance herds (Koketsu 2007), can help break the cycle of reproductive failure and stabilize herd productivity.

Regarding farrowing induction practices, all farms in this study used induction to improve farrowing assistance, either systematically (80% in the low-performance cluster; 58.3% in the high-performance cluster) or selectively for delayed farrowing sows (20% in the low-performance cluster; 41.7% in the high-performance cluster). No significant differences between clusters were observed in this regard (*P* = 0.38). Studies have shown that farrowing induction does not impact SB or PWM when performed two or fewer days before the expected farrowing date, with some reports indicating reduced stillborn risk when induction occurs 1 d before the expected date (Monteiro et al 2022). However, the interaction between farrowing induction and parity has been minimally explored in the literature. Previous research suggests that prostaglandin and/or oxytocin administration for farrowing induction does not significantly affect piglet viability, farrowing process, fetal blood glucose, or hemoglobin levels (Holtz et al 1983; Welp et al 1984; Vallet and Miles 2017; Mills et al 2021). Nonetheless, concerns persist regarding its potential impact on colostrum yield and quality, with some studies indicating negative effects (Jackson et al 1995; Foisnet et al 2011). Due to lower colostrum yield and lower piglet birth weight (Machado et al 2016; Nuntapaitoon et al 2020; Juthamanee et al 2024), avoiding farrowing induction in first parity sows could help mitigate colostrum-related deficiencies, potentially contributing to lower PWM in high-performance clusters.

Creep-feeding practices also differed between clusters, with 75% of high-performing farms initiating creep-feeding by d 5 post-farrowing, compared to only 25% in low-performing herds. While no standardized recommendation exists regarding the optimal age to start creep-feeding, studies suggest initiation can range from as early as 2–3 d to as late as 2–3 d before weaning (Tokach et al 2020; Muro et al 2023). Most comparative analyses focus on feeding intervals within the first or second week of lactation, but early initiation remains underexplored. Given that piglet feed consumption is minimal during the first week of lactation (Tokach et al 2020), this could explain why early creep-feeding is rarely analyzed. The primary documented benefits of creep-feeding involve improving litter weight gain rather than pre-weaning mortality (Muro et al 2023). In the context of our study, early creep-feeding in high-performance farms appears to be more related to improved piglet care than nutritional supplementation. Frequent feed replacement enables workers to observe sows and piglets more regularly, which may contribute to improved piglet survival, enhanced sow performance during lactation, and better reproductive outcomes in subsequent cycles.

Traditionally, herd classification has relied on a single KPI, particularly for piglets weaned per sow per year (PSY). Studies categorizing herds based on PSY report higher FR, increased piglets born alive, and lower PWM in high-performing herds without significant differences in SB rates (Koketsu 2000, 2007). Similarly, herd categorization using piglets born alive shows higher FR and lower parity-related return risks among high-performing herds than ordinary farms (Iida and Koketsu 2015). Our findings align with this, as lower FR in low-performing herds contributes to extended non-productive days, reducing litters per sow per year (Koketsu 2007).

While this study provides valuable insights into herd productivity classification, some limitations should be considered. The number of farms included may influence the ability to detect differences, and the uniformity of management practices within the same production system may limit variability in the factors evaluated. Nevertheless, the results reinforce the effectiveness of unsupervised clustering methods in distinguishing herd performance levels using multiple KPIs. The methodology presented offers a data-driven approach that is adaptable to diverse production systems, enabling the classification of herds and the optimization of management strategies. Additional studies with larger and more varied farm populations could further validate these findings, exploring variations across different farms and pig production systems. Additionally, variables describing other reproductive management practices and parity structure, such as average parity, weaning-to-estrus interval, sows bred within 7 d, and gestation length, should be considered in future studies to better characterize herd reproductive dynamics. Results should be cautiously extrapolated to farms with similar production and health conditions.

## Conclusions

This study demonstrated the effectiveness of an unsupervised clustering approach in classifying breeding herds based on farrowing rate, total number of piglets born and born alive, stillborn, and pre-weaning mortality. The analysis allowed us to identify the cluster with higher performance. Key farm-level factors, such as breeding group size, re-serviced sow inclusion, farrowing induction, and creep-feeding strategies, were identified as characteristics differentiating herd performance.

## Supplementary Material

txag036_Supplementary_Data

## List of abbreviations

KPI: key production indicators
PSY: piglets weaned per sow per year
FR: farrowing rate
TPB: total number of piglets born
TBA: total number of piglets born alive
SB: stillborn
PWM: pre-weaning mortality
