# Supplementary material for: Application of unsupervised learning to cluster swine breeding herds based on key performance indicators in Southern Brazil
Source: Transl Anim Sci. 2026 Mar 25;10:txag036. doi: 10.1093/tas/txag036 (PMC13075986; doi:10.1093/tas/txag036)
Supplement: txag036_Supplementary_Data [file txag036_supplementary_data.docx]

**Table 1S.** Survey used to collect data from sow farms

| **Demographics and general characteristics of the farms** |
| --- |
| Farm name: |
| Evaluation date: |
| Region / Location / State: |
| Farm age: |
| Inventory: |
| Genetic: |
| Other activities on the property include agriculture, poultry, and dairy farming, among others.   - Yes ( ) No ( ) - Which ones? |
| Main activity on the property: |
| Solar orientation (north, south, east, west): send geographic coordinates |
| No. of family members: No. of employees: |
| Batch farrowing   - 1 week ( ) 2 weeks ( ) 3 weeks ( ) 4 weeks ( ) |
| Generator on the property:   - Yes ( ) No ( ) |
| Water tank maintained in the shadow:   - Yes ( ) No ( ) |
| **Labor** |
| Farm labor provided by owners: Yes ( ) No ( ) |
| Total number of employees   - Farrowing: _____________ - Gestation:______________ |
| Number of employees have worked on the farm   - General - under 5 years:_____ over 5 years:_____ - Farrowing – under 5 years:_____ over 5 years:_____ - Gestation – under 5 years:_____ over 5 years:_____ |
| **Gestating infrastructure and handling** |
| Environmental control methods:   - Curtain ( ) Climatized ( ) - Number of exhaust fans: ___ |
| Presence of grass and trees (immediately surrounding the farm):   - Grass: Yes ( ) No ( ) - Trees: Yes ( ) No ( ) |
| Rebreeding of opportunistic females:   - Yes ( ) No ( ) - Target % established: __________ - Percentage observed in records:______ |
| Grouping moment:   - Gestation age? ___ days - Crate ( ) Pen ( ) |
| Bump-feeding:   - Yes ( ) No ( ) - Days of gestation: ___ - Feed amount: ___ kg |
| Automated lighting control   - Yes ( ) No ( ) |
| Gestation drinker type – crate:   - Nipple ________ Trough ________ |
| Floor feeding while in gestation stalls   - Yes ( ) No ( ) |
| Gestating sows housed in pens   - Yes ( ) No ( ) |
| Use of antimicrobials in gilts at the entrance   - Yes ( ) No ( ) |
| Acclimation of gilts at the entrance using some exposure method   - Yes ( ) No ( ) |
| Use of enteric and/or reproductive feedback in gilts   - Yes ( ) No ( ) |
| Use of enteric and/or reproductive feedback in sows   - Yes ( ) No ( ) |
| Percentage of farms using collective pen gestation   - Yes ( ) No ( ) |
| Sows housed in pens based on parity   - Yes ( ) No ( ) |
| Collective pens   - Yes ( ) No ( ) - Number of females per pen: ______ |
| Pen drinkers:   - How many per female: ______ - Model: Trough ( ) Nipple ( ) |
| Gestation flooring – % by area   - Crate: Iron ( ) Plastic ( ) Slatted concrete ( ) - Pen: Slatted ( ) Concrete ( ) |
| Flushing feed for gilts:   - Yes ( ) No ( ) - Average duration: ___ days |
| **Farrowing infrastructure and handling** |
| Number of farrowing rooms:____________ |
| Number of crates per room:_____________ |
| Different types of farrowing barn infrastructure, i.e., different stall types, varying farrowing room sizes, different temperature control systems, etc.   - Yes ( ) No ( ) |
| Presence of grass and trees (immediately surrounding the farm):   - Grass: Yes ( ) No ( ) - Trees: Yes ( ) No ( ) |
| Environmental control methods (Cooling system):   - Curtain ( ) Climatized ( ) - Number of exhaust fans: ___ |
| Environmental control methods (Heating system):   - Yes ( ) No ( ) |
| Farrowing induction:   - Yes ( ) No ( ) - Farrowing induction in gilts: Yes ( ) No ( ) |
| Encourage sows to stand during lactation   - Yes ( ) No ( ) |
| Creep-feeding: Yes ( ) No ( )  • Starts at how many days? _______________ |

Key performance indicators were obtained from the management software used by farms (Agriness S4)
